# Inhibition of Fibroblast Activation Protein‐α Ameliorates Intervertebral Disc Degeneration via Reduced Vascular Invasion in Cartilage Endplate

**DOI:** 10.1111/cpr.70162

**Published:** 2026-01-13

**Authors:** Hao‐Wei Xu, Sheng‐Jie Chang, Shuo Wang, Xiao‐Wei Liu, Shan‐Jin Wang

**Affiliations:** ^1^ Department of Spinal Surgery Shanghai East Hospital, School of Medicine, Tongji University Shanghai China; ^2^ University Hospital Heidelberg Institute of Computation Biomedicine and Center for Infectiology Freiburg Germany; ^3^ Institute of Medical Biometry and Statistics, Faculty of Medicine and Medical Center‐University of Freiburg Freiburg Germany; ^4^ Shanghai Pudong New Area Geriatric Hospital Shanghai China

**Keywords:** angiogenesis, cartilage endplate, fibroblast activation protein‐α, intervertebral disc degeneration, PI3K/Akt/HIF‐1α/VEGFA pathway

## Abstract

Intervertebral disc degeneration (IDD) is a primary cause of low back pain, with the development of new blood vessels being a key pathological feature. Fibroblast activation protein‐alpha (FAP‐α), a member of the Type II serine protease family, possesses dipeptidase and collagenase activities and is closely linked to angiogenesis. Bioinformatics and immunohistochemical analysis revealed elevated FAP‐α expression and increased angiogenesis in degenerated cartilage endplate (CEP). Co‐culture of FAP‐α‐silenced CEP cells or conditioned media with human umbilical vein endothelial cells (HUVECs) demonstrated a reduction in hypoxia‐inducible factor‐α (HIF‐α) levels, vascular endothelial growth factor (VEGF)‐A and PI3K/AKT phosphorylation, which impaired HUVEC migration and tube formation. Conversely, FAP‐α overexpression enhanced angiogenesis via the PI3K/AKT/HIF‐α/VEGF‐A signalling pathway. In rats with IDD induced by lumbar instability, FAP‐α inhibitors reduced angiogenesis and ossification of the CEP, thereby delaying IDD progression associated with CEP degeneration. Genetic deletion of FAP further slowed IDD progression. Collectively, these findings provide compelling evidence that FAP‐α accelerates IDD by promoting angiogenesis, which disrupts disc homeostasis. Targeting FAP‐α may offer a novel therapeutic approach for mitigating IDD.

## Graphical abstract

Schematic illustration showing that FAP‐α promoted angiogenesis, cytokine release and ECM degradation in the CEP degeneration of the intervertebral disc through upregulating the PI3K/Akt/HIF‐1α/VEGFA pathway.

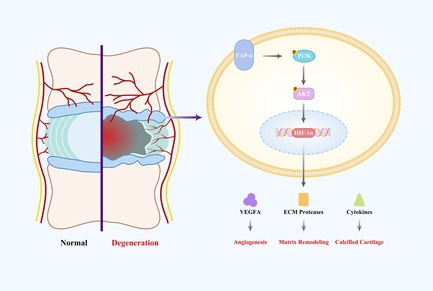

## 1 Introduction

Low back pain has emerged as the leading cause of disability in adults worldwide, placing a substantial economic burden on both society and families. Intervertebral disc degeneration (IDD) is the primary pathological contributor to low back pain [1]. Significant research is underway to explore the mechanisms of IDD, with factors such as age, tissue structure, biomechanics, genetics, inflammation and osteoporosis identified as key modulators of the degenerative process [2]. The adult intervertebral disc (IVD), consisting of the nucleus pulposus (NP), annulus fibrosus (AF) and cartilage endplate (CEP), is the largest avascular tissue in the human body. The NP, a gel‐like substance, is located in a relatively closed environment devoid of blood vessels and nerves. The CEP's diffusion function serves as the primary nutritional supply for the NP. Existing studies suggest that degeneration of the CEP can lead to NP degeneration, thereby accelerating the overall degeneration of the IVD [3].

During IDD progression, microvascular formation is often observed. The formation of microvessels within the IVD correlates with the degree of pathological degeneration. This microvascular development is closely associated with the calcification of the CEP, AF and NP tissues. Calcification further exacerbates nutritional deficiencies and metabolic disruptions within the disc, leading to increased hypoxia, changes in collagen composition and macrophage infiltration [4]. This cascade triggers the upregulation of various biological proteases and cytokines, including tumour necrosis factor, transforming growth factor‐β, VEGF, bone morphogenetic protein and matrix metalloproteinases (MMPs), among others, all of which accelerate the degeneration of the IVD [5].

Fibroblast activation protein‐alpha (FAP‐α), a Type II transmembrane serine protease, plays a pivotal role in the metabolism of various endogenous peptides and peptide‐like drugs. FAP expression is notably higher in tumour tissues compared to normal tissues and contributes significantly to tumour formation, growth and metastasis by enhancing the tumour extracellular matrix (ECM) and microvasculature [6]. Elevated FAP expression has also been observed in inflammatory and fibrotic diseases [7], including wound healing, rheumatoid arthritis, osteoarthritis (OA), liver cirrhosis and pulmonary fibrosis. Zhao et al. [8] demonstrated that FAP overexpression promotes chondrocyte senescence, while genetic knockout of FAP in chondrocytes alleviates OA. Immunohistochemical analysis revealed FAP‐α expression on chondrocyte surfaces in OA cartilage. Given the involvement of pathological angiogenesis and CEP calcification in IDD, a potential link between FAP‐α and IDD exists.

Histological and bioinformatics analyses of CEP tissues from patients with IDD and normal individuals indicate a correlation between FAP‐α expression and IDD progression. It is hypothesized that FAP‐α upregulates the PI3K/AKT pathway, thereby enhancing HIF‐α expression, which subsequently induces VEGF‐A expression and promotes vascular invasion, leading to CEP calcification. To test this hypothesis, various in vitro and in vivo experiments were performed to elucidate the relationships between FAP‐α, the PI3K/AKT pathway, HIF‐α and VEGF‐A, and their roles in IDD progression. The results of this study may shed light on the pathogenic mechanisms of CEP in degenerative disc diseases, offering insights for early repair and therapeutic strategies for IDD.

## 2 Results

### 2.1 FAP‐α Is Upregulated in Human Degenerative Disc Samples and Aged Rats

**FIGURE 1:**
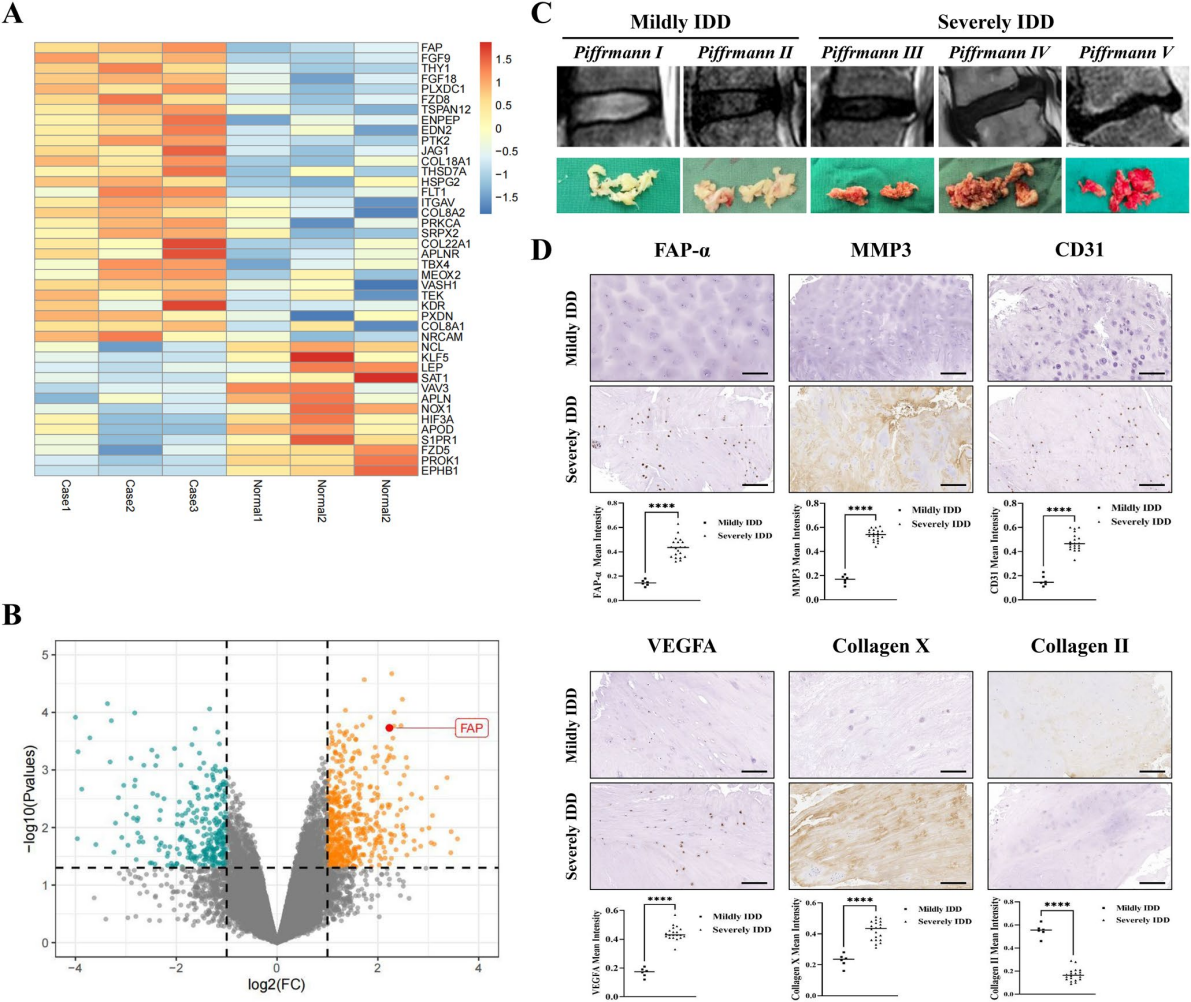
FAP‐α is highly expressed in degenerative cartilage endplate (CEP) tissues. (A) Bioinformatics analysis of the expression profiles from patients with degenerative CEP and healthy controls. Heatmap showing the top differentially expressed genes (DEGs). (B) Volcano plot of DEGs indicating significant changes in mRNA expression (*p* < 0.05). Green dots represent upregulated DEGs, while orange dots represent downregulated DEGs. (C) MRI images and tissue photos of intervertebral discs at different stages of degeneration. (D) Immunohistochemical staining of human CEP tissue for FAP‐α, MMP3, CD31, VEGFA, Collagen X, and Collagen II. Black scale bar = 100 μm. Data are presented as means ± SE, *p < 0.05, **p < 0.01, ***p < 0.001, ****p < 0.0001.

Bioinformatics analyses of gene expression profiles were performed to explore the role of FAP‐α in IDD, comparing samples from patients with CEP degeneration and healthy controls. Differentially expressed genes (DEGs) indicated significant upregulation of FAP‐α in patients with IDD (Figure 1A,B), with a strong association to microvascular invasion and angiogenesis. IVD tissues, representing various degrees of degeneration, were collected via surgical procedures. The baseline data of the patients can be obtained in the Supporting Information. MRI and specimen images of IVDs at each Pfirrmann grade are presented in Figure 1C. These tissues were subsequently used for immunohistochemical staining, revealing that FAP‐α, MMP3, CD31, VEGFA and Collagen X were upregulated, while Collagen II degradation occurred in patients with severe IDD compared to those with mild IDD (Figure 1D).

**FIGURE 2:**
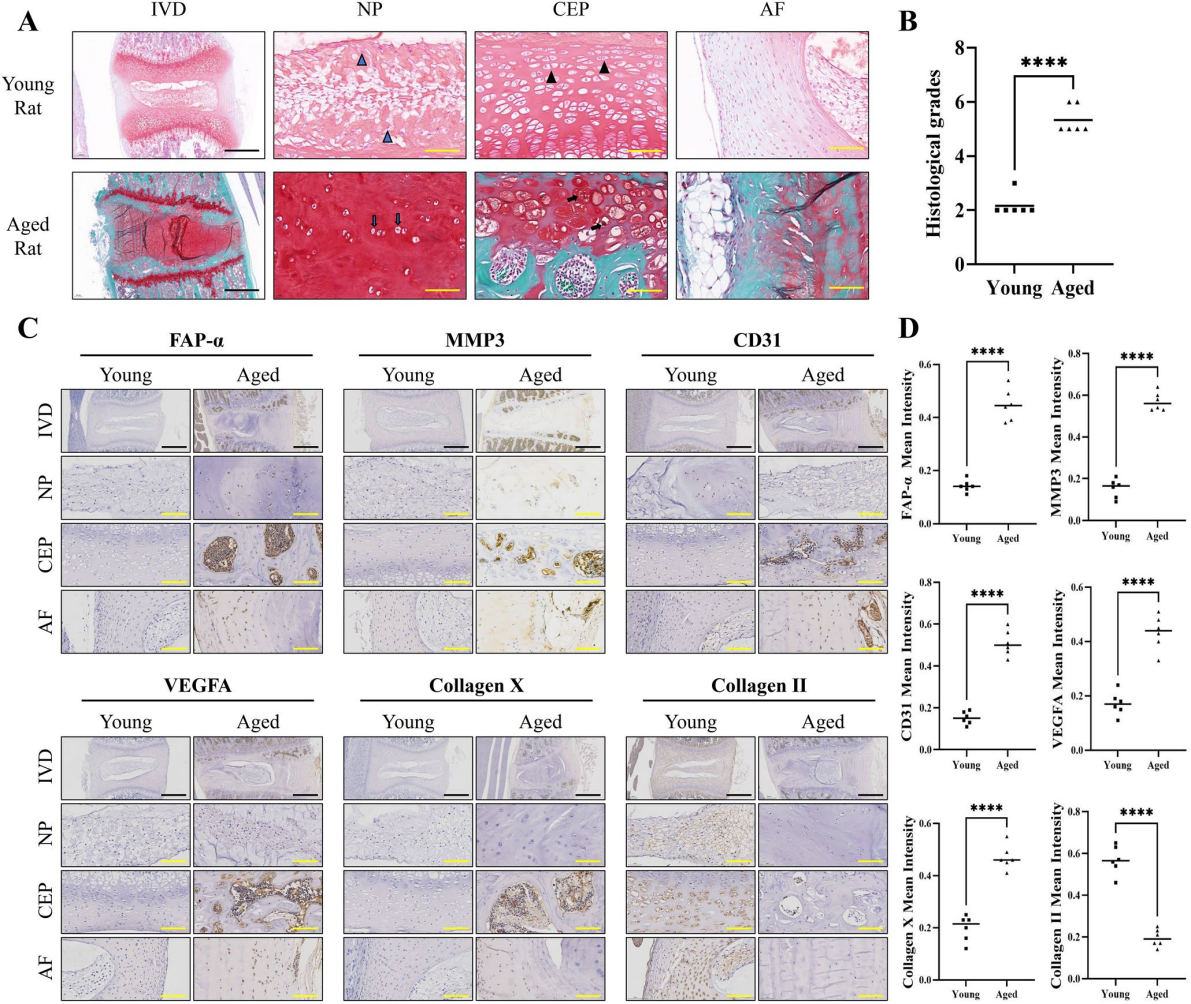
FAP‐α is upregulated in the CEP tissues of aged rats. (A) Representative Safranin O‐Fast Green staining images for each group (female rats, *n* = 6/group). Black scale bar = 200 μm, yellow scale bar = 40 μm. (B) Histological scores of intervertebral discs from rats of different ages. (C, D) IHC detection and quantitative analysis of FAP‐α, MMP3, CD31, VEGFA, Collagen X, and Collagen II. Black scale bar = 200 μm, yellow scale bar = 40 μm. Data are presented as means ± SE, *p < 0.05, **p < 0.01, ***p < 0.001, ****p < 0.0001. AF: annulus fibrosus, CEP: cartilage endplate, IVD: intervertebral disc, NP: nucleus pulposus.

To further validate the relationship between FAP‐α and IDD, a rat model was employed. IVD samples from 1‐month‐old and 2‐year‐old rats were collected and stained with Safranin O‐Fast Green (SO&FG). Notable fissuring of the AF, NP shrinkage or disappearance, CEP calcification and a significantly increased histological score were observed in aged rats (Figure 2A,B), confirming the successful establishment of the age‐related IDD model. Immunohistochemical staining of IVD tissues from both young and aged rats revealed increased expression of FAP‐α, MMP3, CD31, VEGFA and Collagen X, alongside reduced Collagen II expression in the degenerated CEP of aged rats, consistent with the human findings (Figure 2C,D). In conclusion, both human and rat IDD models confirm that FAP‐α upregulation is a prominent feature of IDD, closely associated with matrix degradation and abnormal angiogenesis.

### 2.2 Inhibiting the Expression of FAP‐α Delays the Occurrence of IDD

**FIGURE 3:**
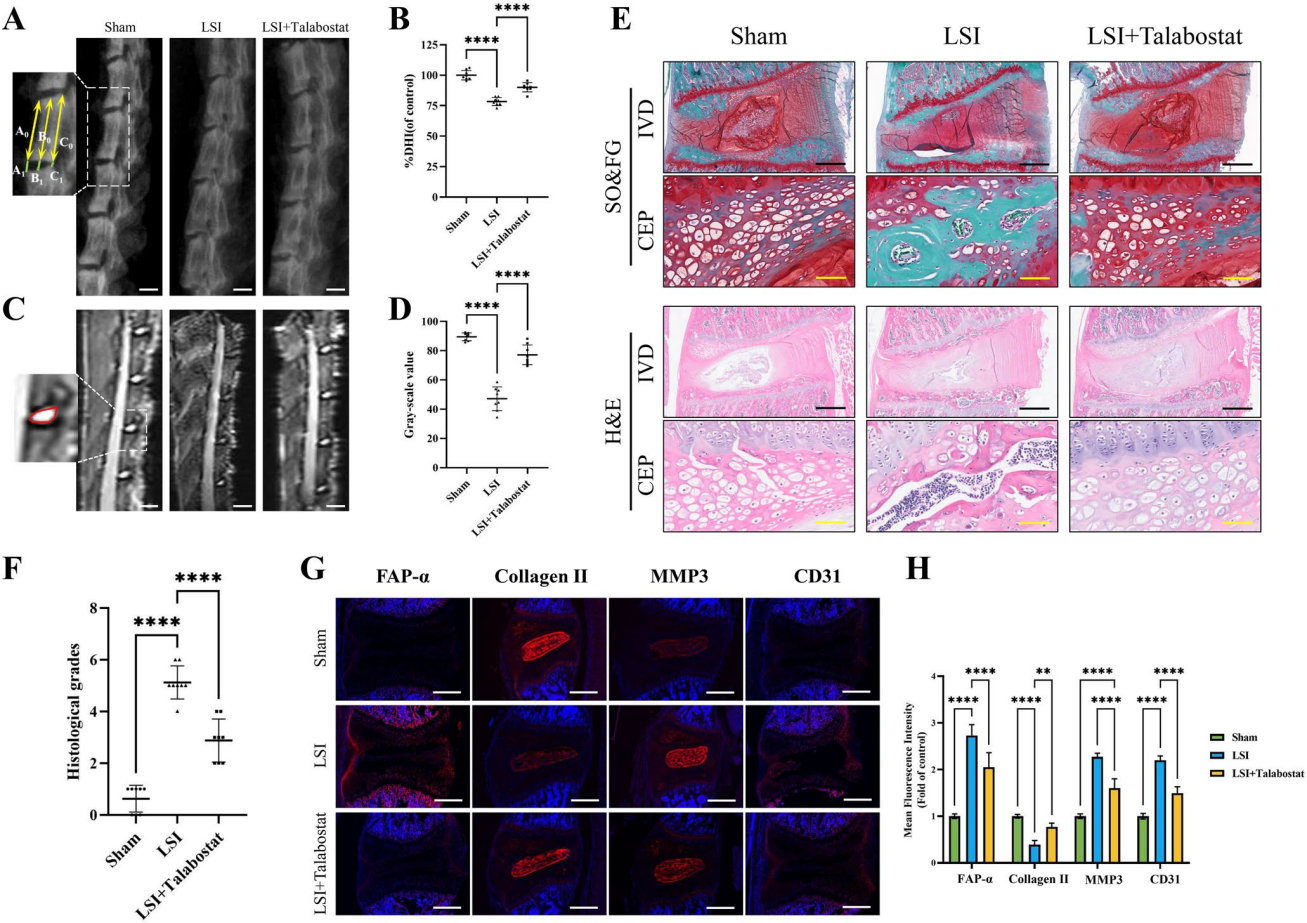
Talabostat ameliorates IDD in the rat lumbar spine instability model 3 months post‐surgery. (A, B) Representative X‐ray images and disc height indices (DHI) of rat intervertebral discs. DHI% was calculated as: DHI% = (A0 + B0 + C0) × 100% / (A0 + B0 + C0 + A1 + B1 + C1). White scale bar = 200 μm, *n* = 8. (C, D) T2‐weighted MRI images of rat lumbar spines from different experimental groups. Grey‐scale values of regions of interest (red irregular circle) in the intervertebral disc (IVD) are shown. White scale bar = 200 μm, *n* = 8. (E, F) Representative Safranin O‐Fast Green and H&E staining of disc samples from different experimental groups with corresponding histological grades. Black scale bar = 200 μm, yellow scale bar = 40 μm. (G, H) Representative immunofluorescence images of FAP‐α, Collagen II, MMP3, and CD31 in intervertebral discs from different experimental groups. White scale bar = 200 μm, *n* = 8. Semi‐quantitative analysis of fluorescence intensity. Averages ± SD, *p < 0.05, **p < 0.01, ***p < 0.001, ****p < 0.0001. CEP: cartilage endplate, IVD: intervertebral disc.

To assess the effects of FAP‐α on IDD progression in vivo, rats were randomly assigned to the Sham, lumbar spine instability (LSI) and LSI + talabostat groups. Twelve weeks post‐surgery, X‐ray imaging revealed decreased intervertebral space height, and MRI scans showed reduced disc signal intensity in the LSI group, confirming the successful establishment of the IDD model (Figure 3A–D). The LSI + talabostat group exhibited significantly better intervertebral space height and disc signal intensity than the LSI group, indicating that FAP‐α inhibition delays IDD progression.

IVD sections from each group were stained with H&E and SO&FG. Histological analysis revealed that in the Sham group, NP cells formed a single‐cell cluster, the AF was uniformly layered and a clear boundary between the AF and NP was evident. In the LSI group, NP cells were markedly reduced, replaced by proliferative fibrous tissue, with occasional mucoid degeneration and concentric circular fissures in the AF, additionally, chondrocyte proliferation into clusters and continuous bone formation in the upper and lower CEP sclerotic bone. In contrast, the talabostat‐treated group showed minimal NP tissue loss, preserved ECM, reduced AF fissuring and clearer AF‐NP boundaries. Histological scores in the LSI + talabostat group were significantly lower than those in the LSI group, demonstrating the therapeutic effect of talabostat (Figure 3E,F).

Immunofluorescence staining revealed a significant increase in FAP‐α expression in the LSI group compared to the Sham group (Figure 3G,H). Following talabostat treatment, FAP‐α levels were significantly reduced. In the talabostat group, Collagen II expression in the NP and CEP increased, while MMP3 expression, which promotes ECM degradation and fibrosis, decreased. CD31 staining, a marker for vascular endothelial cells [9], indicated fewer blood vessels in the CEP of the LSI + talabostat group compared to the LSI group, suggesting reduced angiogenesis following talabostat treatment. These findings indicate that talabostat‐mediated FAP‐α inhibition effectively delays IDD progression by protecting the ECM, reducing ECM degradation and inhibiting abnormal angiogenesis.

### 2.3 Genetic Deletion of FAP Delays IDD Progression

**FIGURE 4:**
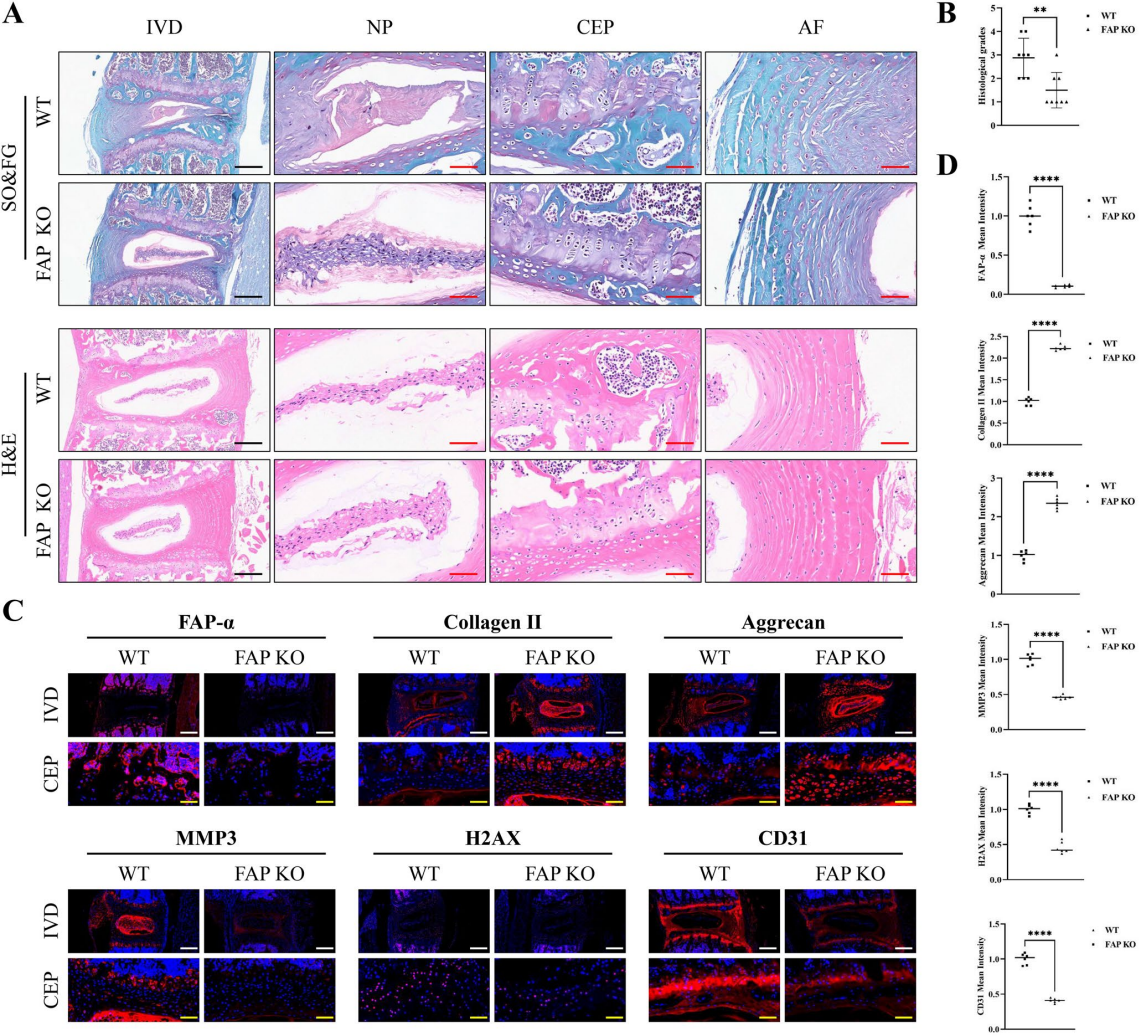
Lumbar intervertebral discs of wild‐type (WT) and FAP KO mice in the ageing model. (A) Representative Safranin O‐Fast Green and H&E staining images of different groups of aged mice. Black scale bar = 200 μm, red scale bar = 40 μm, *n* = 8. (B) Histological scores of intervertebral discs in different groups of aged mice. (C, D) Representative immunofluorescence images of FAP‐α, Collagen II, Aggrecan, MMP3, H2AX, and CD31 in disc samples. White scale bar = 200 μm, yellow scale bar = 40 μm, *n* = 8. Semi‐quantitative analysis of fluorescence intensity. Averages ± SD, *p < 0.05, **p < 0.01, ***p < 0.001, ****p < 0.0001. AF: annulus fibrosus, CEP: cartilage endplate, IVD: intervertebral disc, NP: nucleus pulposus.

FAP knockout (KO) mice were generated and raised alongside wild‐type (WT) mice until 16 months of age to establish a natural ageing IDD model. Histological analysis using H&E and SO&FG staining revealed that, compared to WT mice, the CEP of 16‐month‐old FAP KO mice contained a greater number of chondrocytes, which preserved a longitudinal arrangement (Figure 4A). In the WT group, the CEP exhibited bone marrow cavity tissue, containing haematopoietic cells, osteoclasts and mature vascular tissues (Figure S1). In contrast, the NP of FAP KO mice exhibited abundant notochordal cells with large vacuole‐like structures within their cytoplasm. These cells were tightly arranged in a well‐organised pattern, with clear delineation between the NP cell clusters and surrounding fibrous tissue. The histological degeneration score was significantly lower in the FAP KO mice than in the WT mice (Figure 4B).

In addition, the IVD of FAP KO mice showed significantly increased expression of Collagen II and aggrecan, while the levels of MMP3 and H2AX were reduced (Figure 4C,D). Alongside the FAP knockout, a decrease in CD31 expression was observed in the CEP, suggesting reduced angiogenesis. These findings indicate that genetic deletion of FAP protects the ECM, inhibits its degradation, delays ageing and reduces abnormal blood vessel formation, thereby playing a protective role in the progression of IDD.

### 2.4 FAP‐α Enhances the Angiogenic Activities of HUVECs, Which Exhibit Elevation in P‐p65 and MMP3 Expression Levels

**FIGURE 5:**
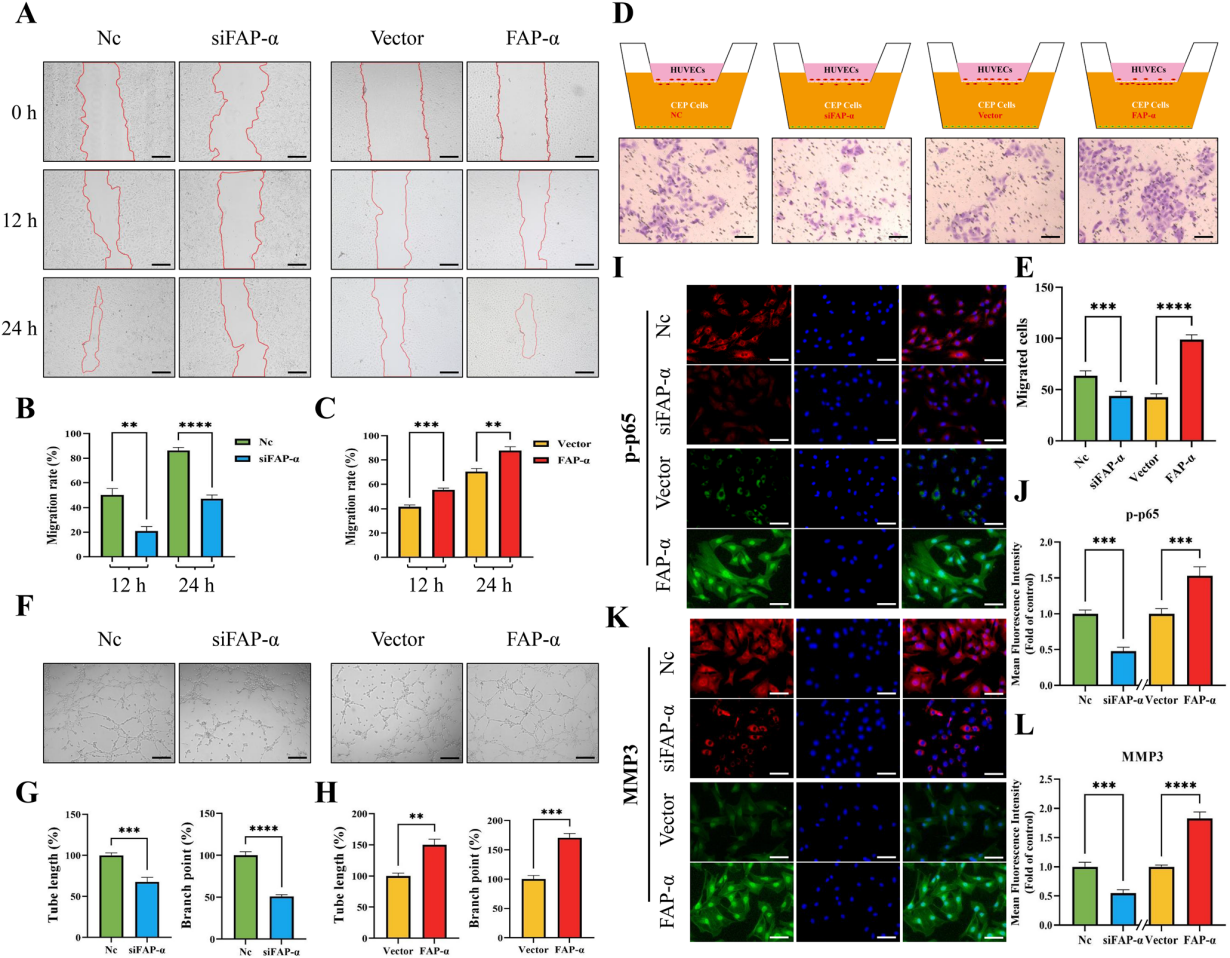
Effects of FAP‐α on blood vessel function. (A‐C) Optical images showing the effect of FAP‐α knockdown or overexpression in conditioned media on HUVEC migration at 24 and 48 h (scale bar = 200 μm). Quantification of migration area (%) by ImageJ. (D‐E) Schematic of the co‐culture experiment of HUVECs and CEP cells, and image of crystal violet‐stained migrating HUVECs (scale bar = 200 μm). (F‐H) Representative images of the tube formation assay on Matrigel in HUVECs treated with FAP‐α conditioned medium (scale bar = 200 μm). Quantitative analysis of the number of branch points (%) and tube length (%). (I‐L) Representative immunofluorescence images of p‐p65 and MMP3 in CEP cells, and semi‐quantitative analysis of fluorescence intensity. Scale bar = 50 μm. Averages ± SD, *p < 0.05, **p < 0.01, ***p < 0.001, ****p < 0.0001.

To examine the impact of conditioned medium from FAP‐α gene‐silenced or overexpressing CEP cells on HUVEC migration, scratch assays were conducted. The conditioned medium from FAP‐α‐silenced CEP cells significantly inhibited HUVEC migration towards the scratch area compared to the untreated CEP cell‐conditioned medium. In contrast, conditioned medium from FAP‐α‐overexpressing CEP cells (achieved by transfection with a plasmid carrying the FAP‐α gene) promoted HUVEC migration towards the scratch area when compared to the Vector group (Figure 5A–C). Similar results were observed in the Transwell assay. Silencing FAP‐α in CEP cells and co‐culturing them with HUVECs led to inhibited HUVEC migration, with a significant decrease in the number of HUVECs that migrated to the lower surface of the polycarbonate membrane in the Transwell chamber. Conversely, FAP‐α overexpression in CEP cells resulted in enhanced HUVEC migration, as confirmed by crystal violet staining (Figure 5D,E). An in vitro angiogenesis assay using a matrix gel model15 showed that the conditioned medium from FAP‐α‐silenced CEP cells suppressed the formation of capillary‐like structures compared to the control group (Figure 5F). Quantitative analysis revealed a reduction in branch points and tube length in the silenced group, whereas conditioned medium from FAP‐α‐overexpressing CEP cells resulted in the opposite effect (Figure 5G,H).

p65, a key regulator of NF‐κB activation and function16, promotes the expression of vascular endothelial growth factor (VEGF)17, while MMPs regulate ECM degradation, both of which are crucial for angiogenesis [10]. Immunofluorescence staining showed that p‐p65 expression was lower in the siFAP‐α group than in the control group, with p‐p65 mainly localised in the cytoplasm (Figure 5I,J). In contrast, in the FAP‐α overexpression group, the distribution of p‐p65 was markedly altered, with enhanced signals throughout the cell and significant accumulation in the nucleus, indicating nuclear translocation. This translocation triggered gene transcription. The expression of MMP3 in each group mirrored the trend of p‐p65 phosphorylation (Figure 5K,L).

Overall, our findings suggest that FAP‐α promotes HUVEC migration and angiogenesis by regulating p‐p65 phosphorylation and MMP3 expression. Upregulation of FAP‐α in CEP cells activates the p65 pathway and upregulates MMP3, thereby enhancing the migration and tube formation abilities of vascular endothelial cells. These results uncover a potential molecular pathway driving pathological angiogenesis during IDD.

### 2.5 FAP‐α Promotes Angiogenesis, Induces Inflammation and Inhibits Matrix Synthesis to Facilitate IDD

**FIGURE 6:**
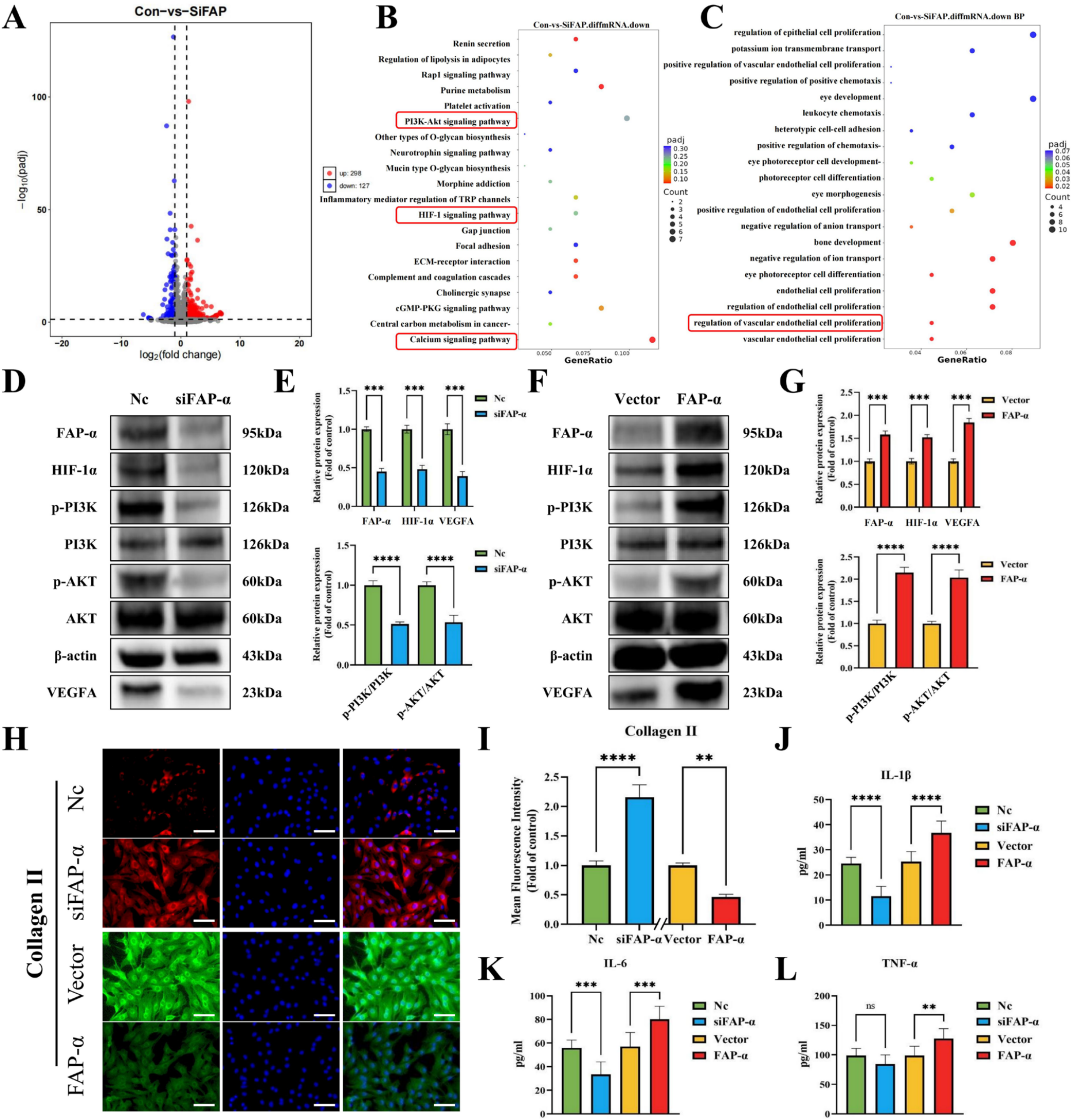
FAP‐α activates the PI3K/Akt/HIF‐1α/VEGFA pathway, increases the release of inflammatory cytokines, and promotes Collagen II degradation. (A) Volcano plot of gene differences between CEP cells with siFAP and the control group. (B) KEGG analysis of differentially expressed gene pathways. (C) GO analysis of differentially expressed gene pathways. (D, E) Western blot results show the protein levels of FAP‐α, HIF‐1α, p‐PI3K, PI3K, p‐AKT, AKT, and VEGFA in the control group and the siFAP‐α group. (F, G) Western blot results show the protein levels of FAP‐α, HIF‐1α, p‐PI3K, PI3K, p‐AKT, AKT, and VEGFA in the Vector group and the overexpression of FAP‐α group. (H, I) Representative immunofluorescence images of Collagen II in CEP cells and semi‐quantitative analysis of fluorescence intensity. Scale bar = 50 μm. (J–L) The levels of IL‐1β, IL‐6, and TNF‐α in supernate of each group were measured using ELSIA. Averages ± SD, *p < 0.05, **p < 0.01, ***p < 0.001, ****p < 0.0001.

To investigate the mechanism by which FAP‐α enhances VEGFA release, RNA sequencing was performed on CEP cells with FAP‐α gene silencing via siRNA and control cells. DEGs were primarily enriched in the PI3K/AKT signalling pathway, the HIF‐1 pathway, calcium signalling and the regulation of vascular endothelial cell proliferation (Figure 6A–C). Western blot analysis confirmed the expression of PI3K, phosphorylated PI3K (p‐PI3K), AKT, phosphorylated AKT (p‐AKT), HIF‐1α and VEGFA. FAP‐α silencing significantly inhibited PI3K/AKT activation and reduced the levels of HIF‐1α and VEGFA compared to the control group (Figure 6D,E). In contrast, FAP‐α overexpression, induced by plasmid transfection, led to enhanced PI3K/AKT activation and increased HIF‐1α and VEGFA expression levels relative to the Vector group (Figure 6F,G). These results confirm that FAP‐α promotes VEGFA expression via the PI3K/Akt/HIF‐1α pathway.

Immunofluorescence staining of Collagen II revealed that FAP‐α knockout in CEP cells significantly upregulated Collagen II expression compared to the control group, while overexpression of FAP‐α significantly reduced Collagen II levels compared to the Vector group (Figure 6H,I), confirming IDD progression. ELISA results further showed that silencing FAP‐α decreased the levels of inflammatory cytokines (IL‐1β and IL‐6) in the culture medium of CEP cells, while FAP‐α overexpression increased the levels of IL‐1β, IL‐6 and TNF‐α (Figure 6J–L). These results suggest that the promotion of VEGFA expression via the PI3K/Akt/HIF‐1α pathway, while a key mechanism for angiogenesis, is not the sole mechanism through which FAP‐α contributes to IDD. Additionally, FAP‐α accelerates degeneration by inhibiting matrix synthesis and promoting inflammatory responses.

## 3 Discussion

IDD is the primary cause of lower back pain, creating a significant economic and social burden globally. The IVDs in adults represent the largest avascular tissues in the human body [11], and extensive studies have demonstrated that pathological blood vessel formation within these discs is a key feature of their degeneration [12]. As IDD progresses, blood vessels and nerves grow into the inner regions of the discs, initiating neurogenic inflammation and the release of mediators such as substance P and other inflammatory factors, which exacerbate lower back pain [13]. This study systematically investigates the central role of FAP‐α in driving IDD progression through clinical specimens, animal models and in vitro experiments. Our findings show that FAP‐α is significantly upregulated in the CEP of degenerated IVDs, with its expression correlating positively with the severity of IDD. Inhibition of FAP‐α function effectively delays the degeneration process. This article delves into the dual molecular mechanisms by which FAP‐α accelerates IDD through the coordinated regulation of angiogenesis and ECM metabolism.

Moreover, FAP‐α activates the PI3K/Akt signalling pathway, stabilising and upregulating the expression of HIF‐1α. In the hypoxic microenvironment of IDD, HIF‐1α, a key transcription factor, directly binds to and promotes the transcription and release of VEGF‐A [14]. Recent studies have highlighted the critical role of HIF‐1α in herniated discs, influencing the survival of IVD cells and the resorption of herniated discs [15]. Wang et al. [16] demonstrated that HIF‐1α gene knockout in mice delayed IDD progression, while aberrant activation of HIF‐1α promotes glycolysis and inhibits mitochondrial function, exacerbating IDD pathology. Thus, regulating abnormal HIF‐1α expression is pivotal in the onset and progression of IDD. FAP‐α is involved in signalling pathways that mediate angiogenesis and promotes the secretion of factors by cells to facilitate blood vessel formation. Harris et al. [17] reported that FAP and VEGFA levels were significantly elevated in patients with endometrial cancer, correlating with tumour vascularization, myometrial invasion and mismatch repair status. Cao et al. [18] found that FAP‐α upregulates VEGFA expression through the Akt and ERK pathways, promoting angiogenesis in colorectal cancer. Zeng et al. [19] reported that in osteosarcoma cells, FAP enhances VEGFA expression and angiogenesis via the AKT and ERK signalling pathways. Our data suggest that FAP‐α similarly drives the formation and ingrowth of blood vessels within the IVD, accelerating degeneration, with this process dependent on the PI3K/AKT pathway.

Notably, while our bioinformatics analysis of human CEP tissue identified FAP‐α as a key driver of angiogenesis, it also revealed the downregulation of several angiogenesis‐related genes in the CEP (Figure 1A). This does not contradict our findings. In the pathological environment of IDD, vascular invasion represents a form of ‘disordered’ or ‘pathological’ angiogenesis. The downregulation of specific pro‐angiogenic genes observed in our analysis does not negate the presence of vascular invasion; rather, it reflects the incomplete and dysfunctional nature of this pathological angiogenesis, closely linked to the unique hypoxic and inflammatory microenvironment of the IVD [20]. Genes such as S1PR1 (which maintains vascular barrier integrity) [21] and PROK1 (which precisely regulates angiogenesis) [22] are typically associated with the formation of stable and functional vascular networks. Their downregulation in degenerated tissue strongly suggests that the invading blood vessels are functionally abnormal, unstable and prone to leakage. This leakage facilitates the infiltration of inflammatory cells and edema, further exacerbating degeneration. Moreover, angiogenesis is a multi‐step process that is tightly regulated by various factors. In IVDD, initial vessel budding may be driven by more potent signals, such as VEGF (which is strongly induced by hypoxia and inflammation) [23], MMPs (which degrade the matrix to allow vessel ingrowth) [24], or inflammatory mediators like TNF‐α and IL‐1β. The downregulated genes identified (e.g., LEP, HIF3A, S1PR1, PROK1) are primarily involved in the later stages of angiogenesis, where the vessels mature and become stable [25]. Their downregulation suggests that angiogenesis in this context is ‘out of control’, leading to the formation of ineffective and harmful blood vessels, a pattern often seen in tumours [26]. In the late stages of IDD, persistent hypoxia and inflammation may trigger a negative feedback mechanism, leading to altered expression of genes such as HIF3A (an inhibitory subunit of HIF). However, this represents a failed compensatory response that cannot reverse the dominant pro‐angiogenic signals mediated by HIF‐1α [27]. In summary, vascular invasion should not be viewed merely as a ‘switch’ event but as a complex and dysfunctional biological process. Our data suggest that while vascular invasion occurs, the resulting vessels are structurally and functionally abnormal, which contributes to tissue destruction rather than repair.

FAP‐α not only promotes the migration of vascular endothelial cells to form blood vessels but also directly contributes to IDD by participating in the degradation and remodelling of the ECM [28]. Collagen II plays a pivotal role in maintaining IVD function. When the content of Collagen II in the ECM of the CEP and NP is reduced, the stress distribution across the disc becomes uneven and its resistance to stretching is diminished, thereby accelerating IDD progression [29]. FAP‐α exists in both membrane‐bound and soluble forms [30]. In its soluble form, it acts as a proline‐specific protease with unique endopeptidase activity [31, 32]. Notably, 15%–30% of the amino acids in Type II collagen are proline [33]. Fan et al. [34] reported that although FAP‐α cannot directly cleave complete natural Collagen II, it forms an efficient ‘degradation alliance’ with MMP1310: MMP13 first makes an initial cleavage on natural collagen, exposing proline‐containing sites and then FAP‐α further degrades them. This sequential action significantly accelerates the structural collapse of the ECM, clearing the path for endothelial cell migration and tubular structure formation. Thus, FAP‐α creates necessary conditions for vascularization by both ‘signal induction’ and ‘matrix remodelling’.

Some functions of FAP‐α may be independent of its enzymatic activity, as it can mediate intracellular signal transduction, modulate the expression of cytokines such as MMPs and indirectly promote ECM degradation [35, 36]. Our research further revealed that FAP‐α overexpression can upregulate the secretion of inflammatory factors like IL‐1β and IL‐6. These cytokines not only directly stimulate MMP expression but also form a positive feedback loop with the NF‐κB pathway, jointly establishing a persistent destructive inflammatory microenvironment for the ECM. In line with our findings, Guan et al. [37] reported that in cancer‐associated fibroblasts, treatment with retinoic acid significantly downregulated FAP expression, inhibited IL‐6 secretion and consequently reduced matrix degradation and tumour cell migration induced by IL‐6.

Additionally, this study observed an intriguing phenomenon in 16‐month‐old FAP KO mice, which retained the characteristics of a young IVD, including a high presence of notochord cells and large vesicular structures within their cytoplasm [38]. In contrast, 16‐month‐old WT mice exhibited bone marrow cavity tissue in the CEP, containing haematopoietic cells, osteoclasts and mature vascular tissue. This complete ‘bone marrow cavity tissue’ appears to be a passive result of FAP‐α‐driven angiogenesis and inflammation. However, whether FAP‐α actively induces osteogenic and haematopoietic differentiation of local mesenchymal stem cells remains an open question that warrants further investigation. During IDD progression, a positive feedback loop may exist between FAP‐α‐mediated new vessel formation, immune cell infiltration and osteoclast activation. It is critical to determine whether the observed protective effect of FAP‐α inhibition arises from its direct suppression of angiogenesis or by disrupting the ‘vascular‐immune‐osteoclast’ cycle, which is a focus of our ongoing research.

Although this study provides robust evidence highlighting the critical role of FAP‐α in IDD, several limitations should be noted: the animal models used (surgically induced and naturally ageing) and the gene knockout model may not fully replicate the extended and complex natural progression of human IDD; the in vitro cell co‐culture system fails to mimic the intricate three‐dimensional structure and biomechanical microenvironment of the disc; furthermore, future studies are needed to correlate FAP‐α expression with patient symptoms, imaging progression and prognosis in larger clinical cohorts to enhance its clinical translational potential.

## 4 Conclusion

In conclusion, this study reveals that FAP‐α is upregulated during the progression of IDD in both human and rat discs. In vivo, genetic deletion of FAP or the use of FAP inhibitors can reduce CEP sclerosis, inhibit blood vessel growth, and ultimately delay IDD. In vitro, FAP‐α influences angiogenesis and cell migration through the PI3K/AKT/HIF‐1α/VEGFA pathway, while also promoting the release of inflammatory factors and ECM degradation in CEP cells. These results suggest that targeting the FAP‐α‐regulated pathway holds promise as a therapeutic strategy for clinical IDD treatment.

## 5 Materials and Methods

### 5.1 Data Collection and Preprocessing

Gene expression data (GSE153761) were retrieved from the Gene Expression Omnibus (GEO) database, comprising six samples: three from patients with cervical spondylotic myelopathy and three from healthy subjects who had undergone anterior cervical discectomy and fusion for cervical fractures. Differential expression analysis was performed using linear models with Empirical Bayes moderation to improve estimate precision. The DEGs were visualised using a Volcano Plot. Gene set enrichment analysis (GSEA) was conducted to identify overrepresented biological processes associated with the DEGs, and Gene Ontology (GO) terms were evaluated for enrichment using Fisher's exact test. Specific biological pathways of interest, such as ‘angiogenesis’, were further investigated by analysing the corresponding DEGs. A co‐expression network was constructed using the Weighted Gene Co‐expression Network Analysis (WGCNA) package. Pearson correlation coefficients between genes were calculated, and an adjacency matrix was created, filtering out weak correlations. A minimum number of nodes criterion was applied to ensure network robustness.

### 5.2 Mice and Genotyping

The Fap knockout (Fap KO) mice (strain #024288; The Jackson Laboratory) on a C57BL/6J background were utilised in this study. This specific model was selected because it carries a targeted replacement of parts of exons 4 and 5 with a LacZ‐neo cassette, which effectively disrupts the Fap gene open reading frame and leads to a null allele. Mice were maintained by crossing heterozygous (Fap+/−) breeders with C57BL/6J wild‐type (WT) mice (#000664) to generate experimental Fap+/+, Fap+/− and Fap−/− littermates, thereby minimising potential confounding effects from genetic background. Genomic DNA was isolated from mouse tail tips. Genotyping was performed by polymerase chain reaction (PCR) using the following primers: 5′‐TTT GGG CCA GGG TTT TCC CAG TCA C‐3′ (common), 5′‐TGG ACA GGG AGG AAG ACA AG‐3′ (wild‐type reverse) and 5′‐GAG GGC AGA GGC TTA GTG TG‐3′ (mutant reverse). The PCR was carried out using Hieff qPCR SYBR Green Master Mix (Low Rox Plus) (11202ES03, Yeasen, China) in a 7500 Thermal Cycler (Applied Biosystems) under the following conditions: initial denaturation at 95°C for 3 min; followed by 35 cycles of 95°C for 30 s, 60°C for 30 s and 72°C for 45 s; with a final extension at 72°C for 5 min. The amplified products were resolved on a 2% agarose gel, yielding bands of 361 bp for the wild‐type allele and 230 bp for the mutant allele.

### 5.3 RNA Sequencing of NP Cells

For the NP cells in both the control group and the siFAP group, RNA was extracted using Trizol. The cDNA products were analysed using an Agilent 2100 Bioanalyzer. Next, the TruSeq Nano DNA Library Prep Kit (Illumina, USA) was employed, and sequencing was conducted on the Illumina HiSeq x‐10 system. The number of reads per gene was calculated using HTSeq. Differential gene expression analysis was performed using the Limma package (version 3.54.2) in R. GSEA v4.0.3 (https://www.gsea‐msigdb.org/gsea/index.jsp) was utilised to analyse differences in Kyoto Encyclopedia of Genes and Genomes (KEGG) pathways between the model group and the DE‐treated group. Differential expression analysis was performed with the Limma package (version 3.54.2) in R, calculating fold changes (FC), with |log_2_FC| > 2 considered statistically significant (*p* < 0.05). The DEGs were further analysed for pathway enrichment using GSEA and the ClusterProfiler package (version 4.6.2).

### 5.4 Patients and Tissue Specimens

IVD samples were obtained from 26 patients at the Spinal Surgery Department who were diagnosed with lumbar disc herniation, spinal stenosis, or lumbar spondylolisthesis (Table S1). All patients underwent lumbar MRI, and disc degeneration was assessed using the Pfirrmann scale [39], which grades degeneration from I to V, corresponding to scores of 1–5. Scores of 1–2 were classified as mildly degenerated discs, while scores of 3–5 were considered severely degenerated. After extraction, the samples were immediately fixed in 4% paraformaldehyde and subsequently embedded in paraffin.

### 5.5 Immunohistochemistry

For immunohistochemical analysis, paraffin sections were deparaffinised, followed by antigen retrieval using 20 × citric acid solution (pH 6.0). Endogenous peroxidases were blocked by incubating the sections in a 3% hydrogen peroxide solution. A blocking step was performed using 3% bovine serum albumin (BSA) at room temperature for 30 min. After removing the blocking solution, primary antibodies for Collagen II (1:200, 28459‐1‐AP, Proteintech), MMP3 (1:200, 17873‐1‐AP, Proteintech), FAP‐α (1:200, 27596‐1‐AP, Proteintech), CD31 (1:200, A19014, ABclonal), VEGFA (1:200, A12303, ABclonal) and Collagen X (1:200, A11645, ABclonal) were applied to the sections. The slides were incubated overnight at 4°C in a humidified chamber. Secondary HRP‐conjugated antibodies corresponding to the species of the primary antibodies were applied, and the sections were incubated for 50 min at room temperature. DAB (3,3′‐diaminobenzidine) was used for colour development, followed by counterstaining of the nuclei. For fluorescence detection, sections were incubated with fluorescent secondary antibodies, and DAPI was used for nuclear staining. The slides were dehydrated and mounted for analysis. The results were analysed using ImageJ software under a brightfield microscope.

### 5.6 Animals and Experimental Design

In vitro, 3‐month‐old female Sprague–Dawley (SD) rats (*n* = 18), 1‐month‐old female SD rats (*n* = 6) and 2‐year‐old female SD rats (*n* = 6) were maintained under controlled conditions (temperature: 23°C ± 2°C, humidity: 50% ± 5%, 12 h light/dark cycle, free access to food and water). The 3‐month‐old rats were randomly assigned to three groups (*n* = 6 each): the sham operation group, the LSI model group and the LSI + FAP‐α suppression treatment group [40] (5 mg/kg talabostat orally once daily, HY‐13233B, MedChemExpress). The LSI procedure was performed as follows [41]: rats were anaesthetised with 3% sodium pentobarbital at a dose of 30 mg/kg. The animals were then placed in a prone position on a fixation plate after shaving and skin sterilisation. A posterior median longitudinal incision was made along the L1‐L6 spinous process according to iliac ridge positioning (flat L6 vertebra). The skin, subcutaneous tissue and lumbar dorsal fascia were incised, and the sacral spinous muscles were separated from the spinous process. The L1‐L6 spinous processes, supraspinous and interspinous ligaments and articular facet joints were resected. After haemostasis, the incision was sutured layer by layer. After 3 months of treatment, the rats were euthanized with pentobarbital.

### 5.7 Imaging Examination

Twelve weeks post‐surgery, the disc height index (DHI%) was calculated from X‐ray images (uDR 588i, United Imaging, Shanghai, PR China) to assess the degree of lumbar degeneration. The DHI% formula is given by: DHI% = (A1 + B1 + C1/A0 + B0 + C0 + A1 + B1 + C1) × 100%, where A1, B1 and C1 represent the average height of the intervertebral space, and A0, B0 and C0 represent the adjacent vertebrae (Figure 3A). A 3.0 T magnetic resonance imaging (MRI) scanner (PHILIPS‐Achieva 3.0 T, The Netherlands) was used to evaluate disc degeneration in rats. The magnetic field strength was set to 45 mT/m, using a spin‐echo pulse sequence. The average grey value of the sagittal T2 image of the L1/L6 disc in each group was measured, with higher grey values indicating greater water content in the disc and lighter degeneration [42] (Figure 3C).

### 5.8 Histological Analysis

After completing all imaging exams, the rats were euthanized, and the L5/L6 IVDs, including parts of the adjacent vertebrae, were carefully extracted. The discs were fixed in 4% paraformaldehyde for 48 h, followed by decalcification in 10% EDTA for 4 weeks. Each specimen was then longitudinally sectioned in the sagittal plane into five 4‐μm‐thick slices. These sections were stained with H&E and Safranin O and examined under a light microscope to evaluate morphological changes in the IVDs. Scoring (0–7 points) was based on the number of notochord cells, the presence of mucoid degeneration, annulus fibrosis fissures and osteophyte formation [43].

### 5.9 Cell Culture

Chondrocytes from the CEP were isolated from rat IVDs by dissecting the endplate cartilage with a scalpel. The tissue was washed three times with PBS and transferred to a 1.5 mL centrifuge tube. Type II collagenase (2 g/L) was added, and the tissue was digested for 4 h at 37°C. Digestion was terminated by adding DMEM/F12 with 10% FBS. After filtering through a sterile 200‐mesh stainless steel sieve, the filtrate containing chondrocytes was transferred to a 25 cm^2^ culture flask for further cultivation.

### 5.10 Inhibition or Overexpression of FAP‐α

For transfection, rat CEP cells were seeded into a six‐well plate. Once the adherent cell density reached approximately 70%, transfection was carried out according to the Lipofectamine 3000 (L3000008, Thermo Fisher SCIENTIFIC) instructions. FAP‐α‐siRNA (Shanghai GenePharma Co. Ltd.) and pcDNA3.1/FAP‐vector (Shanghai GenePharma Co. Ltd.) were separately transfected into the CEP cells. Negative control siRNA (NC‐siRNA) and pcDNA3.1 were co‐transfected as controls. Cells were collected 74 h post‐transfection for further experiments. The siFAP‐α sequence used was 5′‐GCUCCCAGAACCACUUAUATT‐3′.

### 5.11 Immunofluorescence Staining

CEP cells were fixed in precooled formaldehyde at 4°C for 20 min and then washed three times with PBS. The cells were treated with Triton X‐100 and QuickBlock (P0096, P0256, Beyotime) before being incubated overnight at 4°C with primary antibodies. The primary antibodies used were as follows: MMP3 (1:200, 17873‐1‐AP, Proteintech), P‐p65 (1:200, AP0123, ABclonal) and Collagen II (1:200, 28459‐1‐AP, Proteintech). The cells were then incubated with an FITC‐labelled secondary antibody at room temperature for 1 h, with all steps following the addition of the fluorescent secondary antibody carried out under light‐protected conditions. Finally, the cells were stained with DAPI for 3–5 min, washed three times with TBST (3 min each time), and images were collected.

### 5.12 Western Blot

For protein extraction, RIPA lysis buffer containing protease inhibitors (PC101, GRF101, Shanghai Epizyme Biomedical Technology) was added to the cells, followed by ice‐cold lysis, centrifugation and collection of total cell protein. The protein concentration was determined using the BCA protein assay (ZJ101, Shanghai Epizyme Biomedical Technology). After protein denaturation, the target proteins were transferred to a PVDF membrane by SDS‐PAGE gel electrophoresis. The membrane was blocked with 5% skim milk at 37°C for 1 h and then incubated overnight at 4°C with primary antibodies. The primary antibodies included FAP‐α (1:500, 27596‐1‐AP, Proteintech), HIF‐1α (1:1000, 20960‐1‐AP, Proteintech), P‐PI3K (1:1000, T40116, abmart), PI3K (1:1000, T40115, abmart), P‐AKT (1:1000, T40067, abmart), AKT (1:1000, T55561, abmart), VEGFA (1:1000, A12303, ABclonal) and β‐actin (1:1000, AC026, ABclonal). After primary antibody incubation, the membrane was washed three times with TBST for 5 min each. The membrane was then incubated with a secondary antibody specific to the primary antibody species (1:2000) at room temperature for 1 h and washed three times with TBST for 5 min each. Images were captured using chemiluminescence (SQ201, Shanghai Epizyme Biomedical Technology), and band analysis was performed using ImageJ software.

### 5.13 Transwell Migration Assay

HUVECs and CEP cells were co‐cultured using Transwell chambers (8.0 μm pores, 24‐well plates, Yesen). Briefly, FAP‐α knockout or FAP‐α overexpressing CEP cells (1 × 10^4^ cells per well) were seeded into the bottom chamber and cultured in DMEM/Ham's F‐12 medium supplemented with 10% foetal bovine serum for 24 h. HUVECs were then seeded into the upper chamber and cultured in 500 μL serum‐free medium. The upper chamber was placed into the lower chamber, and co‐cultured at 37°C for 24 h. After the incubation, the Transwell chamber was removed, and cells remaining on the surface of the upper chamber filter membrane were gently wiped away with cotton swabs. The cells that migrated to the surface of the lower chamber filter membrane were fixed, washed with PBS and stained with crystal violet. The cells were observed and counted under a microscope, and the number of cells in the upper, lower, left, right and centre fields (× 40) of each filter membrane were recorded. The mean value of these counts was used to calculate cell mobility [44].

### 5.14 Effect of Vascularization

Pre‐cooled Matrigel was added to a 96‐well plate (50 μL per well) and allowed to solidify at 37°C for 1 h. Then, 3 × 10^4^ HUVECs in the logarithmic growth phase were seeded into each well and treated with FAP‐α knockout or FAP‐α overexpressing media. The cells were incubated for 6 h, and capillary‐like structures were observed under a microscope. The tube formation ability was quantified by calculating the total number of branching points and tube length per well.

### 5.15 Scratch Wound Healing Assay

For the wound healing assay, HUVECs were seeded into a 6‐well plate at a density of 5 × 10^5^ cells per well. Once the cells reached confluence, two perpendicular lines were drawn on the plate with a 200 μL pipette tip. The old medium was discarded, and the cells were gently washed with PBS two to three times. FAP‐α overexpressing or FAP‐α knockout media, supplemented with Mitomycin‐C (5 μg/mL; Sigma) to prevent cell proliferation, were added to the corresponding groups. Images of the wound area were taken at 0, 12 and 24 h post‐wounding. Migration area ratios were calculated as previously described [45].

### 5.16 Statistical Analysis

All experiments were performed at least three times. Data analysis was conducted using SPSS 25.0 software. Differences between groups were assessed using *t*‐tests or analysis of variance, with Tukey's multiple comparisons test for post hoc analysis. A *p* value of < 0.05 was considered statistically significant.

## Author Contributions

S.‐J.W. initiated the idea. H.‐W.X. and S.‐J.C. wrote the assay. S.W. did the data analysis. X.‐W.L. supervised and reviewed the manuscript. All authors read and approved the final manuscript.

## Funding

This work was funded by the Youth Scientific Research Cultivation Foundation of Shanghai East Hospital (DFPY2024025), Investigator‐Initiated Clinical Research Project of Pudong New Area Health Commission (2025‐PWDL‐16), Shanghai East Hospital Specialty Development Project (2024‐DFTS‐009) and Specialized Project for Livelihood Research of Public Institutions under the Pudong New Area Science and Technology Development Fund (PKJ2024‐Y35).

## Ethics Statement

This work conformed to the standards of the Declaration of Helsinki, except for registration in a database. Procedures followed animal welfare guidelines set by the US National Institutes of Health. All human and animal experiments in this study were approved by the Ethics Committees of Shanghai East Hospital, Tongji University School of Medicine (EC.D (BG).016.02.1.2022‐045) and written informed consent was obtained from each patient.

## Conflicts of Interest

The authors declare no conflicts of interest.

## Supporting information


**Data S1:** cpr70162‐sup‐0001‐Supinfo.docx.
**Figure S1:** Immunofluorescence staining of CD34, CD45 and TRAP in WT and FAP KO mice.

## Data Availability

The data that support the findings of this study are available from the corresponding author upon reasonable request.

## References

[1] N. N. Knezevic , K. D. Candido , J. W. S. Vlaeyen , J. Van Zundert , and S. P. Cohen , “Low Back Pain,” Lancet 398, no. 10294 (2021): 78–92. 34115979 10.1016/S0140-6736(21)00733-9

[2] S. Kirnaz , C. Capadona , T. Wong , et al., “Fundamentals of Intervertebral Disc Degeneration,” World Neurosurgery 157 (2022): 264–273. 34929784 10.1016/j.wneu.2021.09.066

[3] C. R. Hassan , W. Lee , D. E. Komatsu , and Y. X. Qin , “Evaluation of Nucleus Pulposus Fluid Velocity and Pressure Alteration Induced by Cartilage Endplate Sclerosis Using a Poro‐Elastic Finite Element Analysis,” Biomechanics and Modeling in Mechanobiology 20, no. 1 (2021): 281–291. 32949306 10.1007/s10237-020-01383-8 PMC7897237

[4] E. J. Novais , R. Narayanan , J. A. Canseco , et al., “A New Perspective on Intervertebral Disc Calcification‐From Bench to Bedside,” Bone Research 12, no. 1 (2024): 3. 38253615 10.1038/s41413-023-00307-3 PMC10803356

[5] H. Xu , J. Li , Q. Fei , and L. Jiang , “Contribution of Immune Cells to Intervertebral Disc Degeneration and the Potential of Immunotherapy,” Connective Tissue Research 64, no. 5 (2023): 413–427. 37161923 10.1080/03008207.2023.2212051

[6] M. A. Abd El‐Azeem , M. A. Ali , and S. H. El‐Shorbagy , “Expression of GLUT4 and FAP in Urothelial Bladder Carcinoma: Correlation With Angiogenesis and Clinicopathological Characteristics,” Journal of the Egyptian National Cancer Institute 34, no. 1 (2022): 40. 36155866 10.1186/s43046-022-00145-0 PMC13314236

[7] W. Qi , L. Jin , C. Wu , et al., “Treatment With FAP‐Targeted Zinc Ferrite Nanoparticles for Rheumatoid Arthritis by Inducing Endoplasmic Reticulum Stress and Mitochondrial Damage,” Materials Today Bio 21 (2023): 100702. 10.1016/j.mtbio.2023.100702 PMC10319325 37408696

[8] X. Zhao , J. Lin , M. Liu , et al., “Targeting FAP‐Positive Chondrocytes in Osteoarthritis: A Novel Lipid Nanoparticle siRNA Approach to Mitigate Cartilage Degeneration,” Journal of Nanobiotechnology 22, no. 1 (2024): 659. 39456041 10.1186/s12951-024-02946-y PMC11515236

[9] Z. Zhang , Q. Gan , J. Han , Q. Tao , W. Q. Qiu , and J. A. Madri , “CD31 as a Probable Responding and Gate‐Keeping Protein of the Blood‐Brain Barrier and the Risk of Alzheimer's Disease,” Journal of Cerebral Blood Flow and Metabolism 43, no. 7 (2023): 1027–1041. 37051650 10.1177/0271678X231170041 PMC10291450

[10] J. H. Song , B. Hwang , S. Lyea Park , et al., “IL‐28A/IL‐10Rβ Axis Promotes Angiogenesis via eNOS/AKT Signaling and AP‐1/NF‐κB/MMP‐2 Network by Regulating HSP70‐1 Expression,” Journal of Advanced Research 73 (2025): 247–263. 39127098 10.1016/j.jare.2024.08.013 PMC12225944

[11] S. J. Chang , X. W. Zhang , H. W. Xu , et al., “CeO(2) Nanoparticles Reduce Oxidative Stress and Delay the Degeneration of Intervertebral Disc,” Bioinorganic Chemistry and Applications 2025 (2025): 3399767. 40693044 10.1155/bca/3399767 PMC12277054

[12] Y. Li , T. Zhang , W. Tian , et al., “Loss of TIMP3 Expression Induces Inflammation, Matrix Degradation, and Vascular Ingrowth in Nucleus Pulposus: A New Mechanism of Intervertebral Disc Degeneration,” FASEB Journal 34, no. 4 (2020): 5483–5498. 32107793 10.1096/fj.201902364RR

[13] Y. Wang , M. Che , J. Xin , Z. Zheng , J. Li , and S. Zhang , “The Role of IL‐1β and TNF‐α in Intervertebral Disc Degeneration,” Biomedicine & Pharmacotherapy 131 (2020): 110660. 32853910 10.1016/j.biopha.2020.110660

[14] Z. R. Schoepflin , E. S. Silagi , I. M. Shapiro , and M. V. Risbud , “PHD3 Is a Transcriptional Coactivator of HIF‐1α in Nucleus Pulposus Cells Independent of the PKM2‐JMJD5 Axis,” FASEB Journal 31, no. 9 (2017): 3831–3847. 28495754 10.1096/fj.201601291R PMC5572688

[15] R. He , Z. Wang , M. Cui , et al., “HIF1A Alleviates Compression‐Induced Apoptosis of Nucleus Pulposus Derived Stem Cells via Upregulating Autophagy,” Autophagy 17, no. 11 (2021): 3338–3360. 33455530 10.1080/15548627.2021.1872227 PMC8632345

[16] Z. Wang , H. Chen , Q. Tan , et al., “Inhibition of Aberrant Hif1α Activation Delays Intervertebral Disc Degeneration in Adult Mice,” Bone Research 10, no. 1 (2022): 2. 34983922 10.1038/s41413-021-00165-x PMC8727577

[17] H. J. Harris , P. Łaniewski , H. Cui , D. J. Roe , D. M. Chase , and M. M. Herbst‐Kralovetz , “Cervicovaginal Lavages Uncover Growth Factors as Key Biomarkers for Early Diagnosis and Prognosis of Endometrial Cancer,” Molecular Biomedicine 5, no. 1 (2024): 55. 39511039 10.1186/s43556-024-00219-6 PMC11543965

[18] F. Cao , S. Wang , H. Wang , and W. Tang , “Fibroblast Activation Protein‐α in Tumor Cells Promotes Colorectal Cancer Angiogenesis via the Akt and ERK Signaling Pathways,” Molecular Medicine Reports 17, no. 2 (2018): 2593–2599. 29207091 10.3892/mmr.2017.8155

[19] C. Zeng , M. Wen , and X. Liu , “Fibroblast Activation Protein in Osteosarcoma Cells Promotes Angiogenesis via AKT and ERK Signaling Pathways,” Oncology Letters 15, no. 4 (2018): 6029–6035. 29552230 10.3892/ol.2018.8027 PMC5840686

[20] Y. Huang , H. Li , L. Qi , et al., “NanoCRISPR‐Assisted Biomimetic Tissue‐Equivalent Patch Regenerates the Intervertebral Disc by Inhibiting Endothelial‐To‐Mesenchymal Transition,” Biomaterials 322 (2025): 123404. 40398216 10.1016/j.biomaterials.2025.123404

[21] H. Zheng , J. Yu , L. Gao , et al., “S1PR1‐Biased Activation Drives the Resolution of Endothelial Dysfunction‐Associated Inflammatory Diseases by Maintaining Endothelial Integrity,” Nature Communications 16, no. 1 (2025): 1826. 10.1038/s41467-025-57124-x PMC11842847 39979282

[22] N. Alfaidy , P. Hoffmann , H. Boufettal , et al., “The Multiple Roles of EG‐VEGF/PROK1 in Normal and Pathological Placental Angiogenesis,” BioMed Research International 2014 (2014): 451906. 24955357 10.1155/2014/451906 PMC4052057

[23] M. He , J. Pang , H. Sun , G. Zheng , Y. Lin , and W. Ge , “P14ARF Inhibits Regional Inflammation and Vascularization in Intervertebral Disc Degeneration by Upregulating TIMP3,” American Journal of Physiology. Cell Physiology 318, no. 4 (2020): C751–c761. 32023075 10.1152/ajpcell.00271.2019

[24] H. J. Moon , T. Yurube , T. P. Lozito , et al., “Effects of Secreted Factors in Culture Medium of Annulus Fibrosus Cells on Microvascular Endothelial Cells: Elucidating the Possible Pathomechanisms of Matrix Degradation and Nerve in‐Growth in Disc Degeneration,” Osteoarthritis and Cartilage 22, no. 2 (2014): 344–354. 24361793 10.1016/j.joca.2013.12.008 PMC3952937

[25] S. Payne , A. Neal , and S. De Val , “Transcription Factors Regulating Vasculogenesis and Angiogenesis,” Developmental Dynamics 253, no. 1 (2024): 28–58. 36795082 10.1002/dvdy.575 PMC10952167

[26] S. Goel , D. G. Duda , L. Xu , et al., “Normalization of the Vasculature for Treatment of Cancer and Other Diseases,” Physiological Reviews 91, no. 3 (2011): 1071–1121. 21742796 10.1152/physrev.00038.2010 PMC3258432

[27] X. Bao , J. Zhang , G. Huang , et al., “The Crosstalk Between HIFs and Mitochondrial Dysfunctions in Cancer Development,” Cell Death & Disease 12, no. 2 (2021): 215. 33637686 10.1038/s41419-021-03505-1 PMC7910460

[28] M. H. Fan , Q. Zhu , H. H. Li , et al., “Fibroblast Activation Protein (FAP) Accelerates Collagen Degradation and Clearance From Lungs in Mice,” Journal of Biological Chemistry 291, no. 15 (2016): 8070–8089. 26663085 10.1074/jbc.M115.701433 PMC4825011

[29] F. Liu , S. Chao , L. Yang , et al., “Molecular Mechanism of Mechanical Pressure Induced Changes in the Microenvironment of Intervertebral Disc Degeneration,” Inflammation Research 73, no. 12 (2024): 2153–2164. 39379638 10.1007/s00011-024-01954-w

[30] J. M. Robbins , P. Rao , S. Deng , et al., “Plasma Proteomic Changes in Response to Exercise Training Are Associated With Cardiorespiratory Fitness Adaptations,” JCI Insight 8, no. 7 (2023): e165867. 37036009 10.1172/jci.insight.165867 PMC10132160

[31] A. J. Lay , H. E. Zhang , G. W. McCaughan , and M. D. Gorrell , “Fibroblast Activation Protein in Liver Fibrosis,” Front Biosci (Landmark Ed) 24, no. 1 (2019): 1–17. 30468644 10.2741/4706

[32] J. Cai , D. Yang , H. Sun , et al., “A Multifactorial Analysis of FAP to Regulate Gastrointestinal Cancers Progression,” Frontiers in Immunology 14 (2023): 1183440. 37325617 10.3389/fimmu.2023.1183440 PMC10262038

[33] A. Steplewski , H. Ito , E. Rucker , et al., “Position of Single Amino Acid Substitutions in the Collagen Triple Helix Determines Their Effect on Structure of Collagen Fibrils,” Journal of Structural Biology 148, no. 3 (2004): 326–337. 15522781 10.1016/j.jsb.2004.07.006

[34] A. Fan , G. Wu , J. Wang , et al., “Inhibition of Fibroblast Activation Protein Ameliorates Cartilage Matrix Degradation and Osteoarthritis Progression,” Bone Research 11, no. 1 (2023): 3. 36588124 10.1038/s41413-022-00243-8 PMC9806108

[35] H. Swahn , K. Li , T. Duffy , et al., “Senescent Cell Population With ZEB1 Transcription Factor as Its Main Regulator Promotes Osteoarthritis in Cartilage and Meniscus,” Annals of the Rheumatic Diseases 82, no. 3 (2023): 403–415. 36564153 10.1136/ard-2022-223227 PMC10076001

[36] R. Bughda , P. Dimou , R. R. D'Souza , and A. Klampatsa , “Fibroblast Activation Protein (FAP)‐Targeted CAR‐T Cells: Launching an Attack on Tumor Stroma,” ImmunoTargets and Therapy 10 (2021): 313–323. 34386436 10.2147/ITT.S291767 PMC8354246

[37] J. Guan , H. Zhang , Z. Wen , et al., “Retinoic Acid Inhibits Pancreatic Cancer Cell Migration and EMT Through the Downregulation of IL‐6 in Cancer Associated Fibroblast Cells,” Cancer Letters 345, no. 1 (2014): 132–139. 24334138 10.1016/j.canlet.2013.12.006

[38] Y. Gan , J. He , J. Zhu , et al., “Spatially Defined Single‐Cell Transcriptional Profiling Characterizes Diverse Chondrocyte Subtypes and Nucleus Pulposus Progenitors in Human Intervertebral Discs,” Bone Research 9, no. 1 (2021): 37. 34400611 10.1038/s41413-021-00163-z PMC8368097

[39] C. W. Pfirrmann , A. Metzdorf , M. Zanetti , J. Hodler , and N. Boos , “Magnetic Resonance Classification of Lumbar Intervertebral Disc Degeneration,” Spine (Phila Pa 1976) 26, no. 17 (2001): 1873–1878. 11568697 10.1097/00007632-200109010-00011

[40] M. A. Sánchez‐Garrido , K. M. Habegger , C. Clemmensen , et al., “Fibroblast Activation Protein (FAP) as a Novel Metabolic Target,” Molecular Metabolism 5, no. 10 (2016): 1015–1024. 27689014 10.1016/j.molmet.2016.07.003 PMC5034526

[41] S. Liu , Y. Sun , J. Dong , and Q. Bian , “A Mouse Model of Lumbar Spine Instability,” Journal of Visualized Experiments 170 (2021): e61722. 10.3791/61722 33970135

[42] H. W. Xu , X. Y. Fang , X. W. Liu , et al., “α‐Ketoglutaric Acid Ameliorates Intervertebral Disk Degeneration by Blocking the IL‐6/JAK2/STAT3 Pathway,” American Journal of Physiology Cell Physiology 325, no. 4 (2023): C1119–c1130. 37661920 10.1152/ajpcell.00280.2023

[43] S. J. Chang , H. W. Xu , S. B. Zhang , X. W. Liu , Y. Y. Yi , and S. J. Wang , “Excessive Cholesterol Accelerates Intervertebral Disc Degeneration by Promoting the Polarization of M1 Macrophages,” Lipids in Health and Disease 24, no. 1 (2025): 305. 41034870 10.1186/s12944-025-02709-0 PMC12490159

[44] C. Y. Chen , S. S. Rao , L. Ren , et al., “Exosomal DMBT1 From Human Urine‐Derived Stem Cells Facilitates Diabetic Wound Repair by Promoting Angiogenesis,” Theranostics 8, no. 6 (2018): 1607–1623. 29556344 10.7150/thno.22958 PMC5858170

[45] X. Li , X. Xie , W. Lian , et al., “Exosomes From Adipose‐Derived Stem Cells Overexpressing Nrf2 Accelerate Cutaneous Wound Healing by Promoting Vascularization in a Diabetic Foot Ulcer Rat Model,” Experimental & Molecular Medicine 50, no. 4 (2018): 1–14. 10.1038/s12276-018-0058-5 PMC5938041 29651102

