# Supplementary material for: Inhibition of Fibroblast Activation Protein‐α Ameliorates Intervertebral Disc Degeneration via Reduced Vascular Invasion in Cartilage Endplate
Source: Cell Prolif. 2026 Jan 13;59(6):e70162. doi: 10.1111/cpr.70162 (PMC13241832; doi:10.1111/cpr.70162)
Supplement: Supplementary file 1 — Data S1: cpr70162‐sup‐0001‐Supinfo.docx. Figure S1: Immunofluorescence staining of CD34, CD45 and TRAP in WT and FAP KO mice. [file CPR-59-e70162-s001.docx]

**Supplementary Information**

**Inhibition of Fibroblast Activation Protein-α Ameliorates Intervertebral Disc Degeneration *via* Reduced Vascular Invasion in Cartilage Endplate**

Hao-Wei Xu^1^, PhD, Sheng-Jie Chang ^1^, MD, Shuo Wang^2,3^, PhD, Xiao-Wei Liu^1^, MD, Shan-Jin Wang^1,4^[[1]](#footnote-1)^*^ MD, PhD.

1. Department of Spinal Surgery, Shanghai East Hospital, School of Medicine, Tongji University, Shanghai, 200092, China.

2. University Hospital Heidelberg Institute of Computation Biomedicine and Center for Infectiology, Freiburg 79098, Germany.

3. Institute of Medical Biometry and Statistics, Faculty of Medicine and Medical Center-University of Freiburg, Freiburg 79098, Germany.

4. Shanghai Pudong New Area Geriatric Hospital, Shanghai 201314, China

Hao-Wei Xu and Sheng-Jie Chang are co-first authors.

***** Corresponding Author: **Shan-Jin Wang**

150# Jimo RD, Pudong New Area, 200120, Shanghai, China

Ph: 86-21-38804518 ext 12025. Fax: 86-21-63595958

Email: [kingspine@163.com](mailto:kingspine@163.com)

**1. Supplementary table**

| Patient ID | Age (Year) | Sex | BMI (kg/m^2^) | Diseased segment | Operational types |
| --- | --- | --- | --- | --- | --- |
| 1 | 62 | Male | 25.2 | L2/3 | TLIF |
| 2 | 28 | Female | 20.2 | L3/4 | PLIF |
| 3 | 63 | Male | 25.5 | L5/S1 | PLIF |
| 4 | 31 | Female | 27.3 | L4/5 | PLIF |
| 5 | 61 | Female | 31.1 | L4/5 | PLIF |
| 6 | 82 | Female | 23.2 | L4/5 | PLIF |
| 7 | 67 | Male | 25.7 | L4/5 | TLIF |
| 8 | 66 | Male | 25.3 | L5/S1 | TLIF |
| 9 | 38 | Male | 23.1 | L5/S1 | TLIF |
| 10 | 69 | Male | 24.6 | L5/S1 | PLIF |
| 11 | 79 | Male | 23.1 | L4/5 | PLIF |
| 12 | 21 | Male | 21.5 | L4/5 | PLIF |
| 13 | 35 | Male | 25.5 | L3/4 | TLIF |
| 14 | 62 | Female | 26.8 | L3/4 | TLIF |
| 15 | 75 | Female | 24.3 | L4/5 | PLIF |
| 16 | 54 | Male | 23.8 | L5/S1 | PLIF |
| 17 | 22 | Female | 27.2 | L5/S1 | PLIF |
| 18 | 58 | Female | 24.1 | L3/4 | PLIF |
| 19 | 57 | Male | 22.7 | L3/4 | TLIF |
| 20 | 58 | Male | 25.1 | L3/4 | PLIF |
| 21 | 45 | Female | 25.2 | L5/S1 | PLIF |
| 22 | 54 | Female | 26.2 | L5/S1 | PLIF |
| 23 | 38 | Female | 19.3 | L5/S1 | PLIF |
| 24 | 58 | Male | 25.8 | L5/S1 | PLIF |
| 25 | 57 | Male | 26.0 | L4/5 | PLIF |
| 26 | 58 | Female | 23.9 | L4/5 | TLIF |

Transforaminal Lumbar Interbody Fusion, TLIF. Posterior Lumbar Interbody Fusion, PLIF.

**2. Supplementary figure**


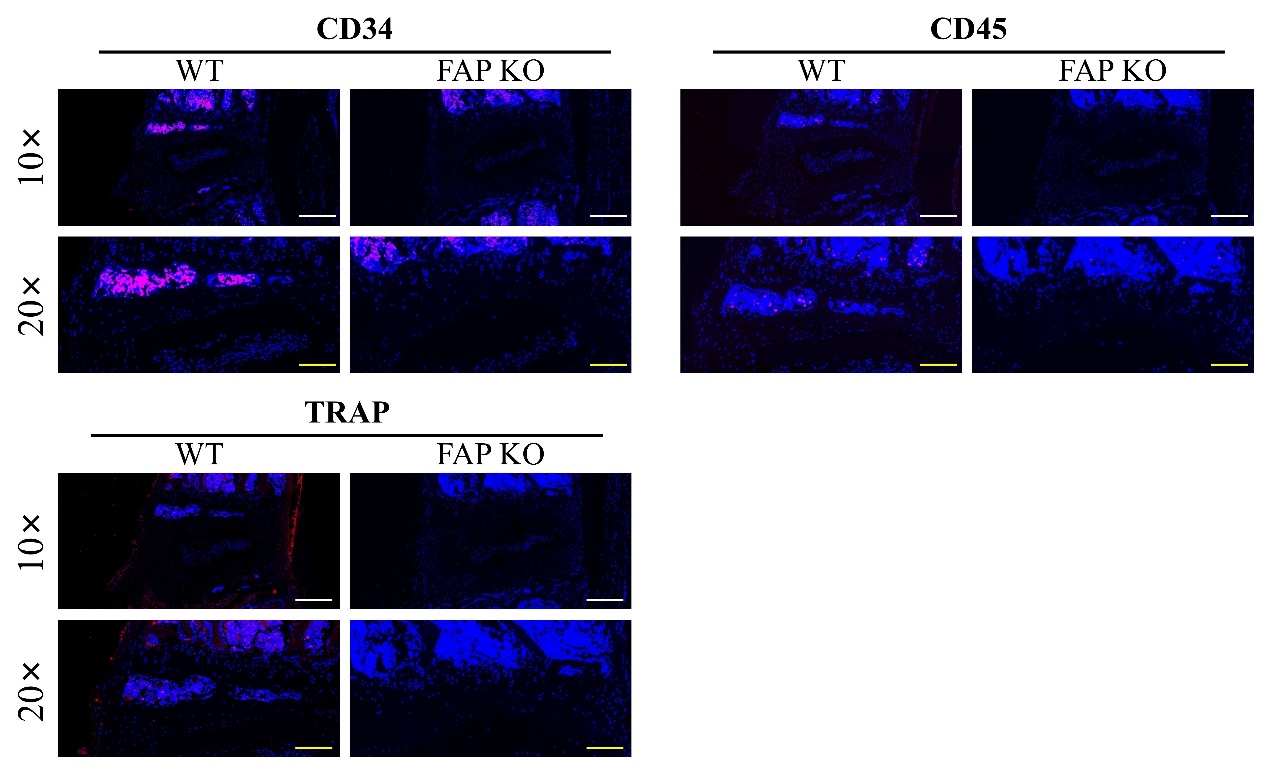


Fig.S1 Immunofluorescence staining of CD34, CD45 and TRAP in WT and FAP KO mice.

1. [↑](#footnote-ref-1)
